# Mind the (gender pay) gap: the role of board gender composition

**DOI:** 10.1007/s00148-026-01159-x

**Published:** 2026-02-21

**Authors:** Yannis Galanakis, Amanda Gosling

**Affiliations:** 1 Economic Statistics Centre of Excellence, King’s College London, King’s Business School, King’s College London, first floor of Melbourne House, Aldwych, London, WC2B 4LL United Kingdom; 2 The Productivity Institute, Alliance Manchester Business School, Manchester, United Kingdom; 3 GLO, Essen, Germany; 4 University of Kent, Canterbury, England

**Keywords:** Board of directors, Women on boards, Gender pay gap, C26, G30, G34, J16, J31, L16

## Abstract

We study how the share of female directors affects company-reported gender pay gaps using linked administrative data. We combine gender pay gap reports with proprietary board-composition data for 8,411 UK firms with at least 250 employees (2017–2021). Our identification uses a Bartik (1991)-style instrumental variable design that exploits regional shifts in female board representation. A one-percentage-point increase in the female director share reduces the gender pay gap by 0.043 percentage points. Moving from the current UK average to board gender parity would close about one-sixth of the reported 9.7% pay gap. The effect operates through three channels. Female directors generate asymmetric wage increases favouring women, improve female representation across pay quartiles, and ensure more equitable allocation of performance-related pay. Effects are concentrated in firms with 250-5,000 employees and are strongest when UK nationals comprise a board majority.

**Supplementary Information:**

The online version contains supplementary material available at 10.1007/s00148-026-01159-x.

## Introduction

Despite decades of progress in female educational attainment and workforce participation (Olivetti and Petrongolo 2016), women in large UK companies earn 10.5% less than their male counterparts for doing the same work.^1^ This persistent, albeit slow-converging, gender pay gap (GPG) represents not just a fairness issue, but a fundamental inefficiency in how firms allocate and reward talent (Blau and Kahn 2017; Goldin 2014). While extensive research has documented individual and workplace factors driving these disparities (Costa Dias et al. 2020; Cukrowska-Torzewska and Lovasz 2016; Manning and Swaffield 2008), a critical question remains underexplored: Can those who oversee corporate governance and shape compensation policies — i.e., the board of directors — influence pay equity throughout their organizations?

This paper examines the impact of the corporate board gender composition on the company-reported GPG in the UK context. In the UK’s unitary (one-tier) corporate governance system, all directors, both executive and non-executive, are collectively responsible for strategy, oversight, and remuneration policies (Hopt and Leyens 2023; Financial Reporting and Council 2018). Boards, therefore, sit close to the levers that shape internal pay setting and evaluation. If board gender diversity improves monitoring or changes priorities, it could narrow company-reported GPGs.

Our focus on boards is theoretically motivated by three complementary frameworks. First, Agency Theory suggests that diverse boards enhance monitoring and reduce managerial discretion that may perpetuate discriminatory practices (Adams and Ferreira 2009). If discrimination represents an ‘agency cost’, namely an inefficient allocation of talent that risks legal and/or reputational penalties, stronger oversight by gender-diverse boards should push towards a more merit-based compensation. Second, Resource Dependence Theory posits that female directors bring unique resources, networks, and legitimacy that can influence HR policies and pay practices (Hillman et al. 2007). Third, Social Role Theory suggests that female leaders may challenge traditional gender stereotypes and advocate for more equitable treatment of women throughout the organization (Eagly and Karau 2002).

To test these theoretical predictions, we exploit two data sources. First, we use the annual gender pay statistics that employers with at least 250 employees report after the UK’s 2017 mandatory pay transparency regime. Second, we collect firm-level board composition and financial details from Moody’s proprietary data. This creates a large-scale panel dataset (8,411 firms, 2017-2021) linking director demographics to firm-level GPG. Our primary outcome is the company-reported GPG in median hourly ordinary pay, excluding bonuses and stock compensation due to data limitations. Our identification strategy addresses the fundamental endogeneity that firms appointing female directors may differ systematically from those that do not. We employ a Bartik (1991)-style shift-share instrumental variable that leverages regional variation in female board representation, as in Flabbi et al. (2019). The instrument is exogenous to firm-specific wage-setting decisions, but correlated with the share of female directors at the firm level.

Our contribution is threefold. First, we provide evidence on board-level gender spillovers in a major economy without binding quotas, extending beyond the management-level effects documented by Theodoropoulos et al. (2022) and Zimmermann (2022). This demonstrates that voluntary diversity can influence organisational outcomes. Second, we identify specific channels through which female directors affect pay equity: differential wage effects favouring women; improved female representation across the pay distribution; and more equitable allocation of performance pay. Third, we document important heterogeneity. Effects are concentrated in mid-sized and large firms (250-5,000 employees) and dominate only when UK nationals constitute the majority of the boardroom.

We find that female directors reduce GPG, though the effect is economically modest. A one percentage point increase in the share of female directors decreases the GPG by 0.043 percentage points. While small in magnitude, this masks important mechanisms. Female directors generate asymmetric wage effects. They raise female wages by 0.11% versus 0.07% male wages per percentage point increase in female board share. They improve female representation not just at the top (vertical spillover) but across all pay quartiles (horizontal spillover). They also ensure more equitable bonus allocation, with the gender gap in performance pay receipt narrowing consistently in firms with female directors. These effects vary importantly by firm characteristics. The impact is strongest in mid-sized and large firms. It disappears in the largest organizations (5,000+ employees), possibly due to more rigid pay structures. Board nationality matters, too. Effects manifest only when UK nationals comprise over 51% of directors, suggesting local knowledge and networks are essential for implementing equity-enhancing practices.

While a growing literature examines gender spillovers from female leadership, most studies focus on management rather than boards, and evidence remains mixed. At the management level, some studies find positive spillovers: female managers reduce gender gaps among subordinates (Theodoropoulos et al. 2022 for the UK, Sondergeld and Wrohlich 2023 and Zimmermann 2022 for Germany, Hensvik 2014 for Sweden), improve women’s promotion prospects (Kunze and Miller 2017; Matsa and Miller 2011), and implement fairer evaluation practices (Abendroth et al. 2017). However, others document limited or even negative effects, particularly the “queen bee syndrome” where female leaders in male-dominated environments distance themselves from other women (Srivastava and Sherman 2015; Bednar and Gicheva 2014).

At the board level, the evidence is even more limited. While studies document that female directors improve firm performance (Post and Byron 2015; Adams and Ferreira 2009) and reduce risk-taking (Sila et al. 2016), few examine their impact on employee outcomes. In Germany, top (or first-tier) management has a non-linear, but modest, impact on GPG (Sondergeld and Wrohlich 2023; Zimmermann 2022). Notable exceptions include Bertrand et al. (2019), who find limited spillovers from Norway’s board quota, and Maida and Weber (2022), who document positive effects from Italy’s board reform. Kirsch (2022) specifically examines how female directors act as “change agents” for gender equality within organizations. Recent work by Von Essen and Smith (2024) finds network effects from female directors in Denmark, while earlier studies show mixed evidence on whether board diversity affects women’s representation in management (Bozhinov et al. 2021; Gould et al. 2018). Related work on directors of foreign nationality documents both benefits and local monitoring frictions in host countries (e.g., Masulis et al. 2012; Ahamed et al. 2019).

No study has leveraged the UK’s unique institutional setting to identify causal effects of female directors on company-reported GPG. The UK combines mandatory pay transparency with voluntary board diversity initiatives, which differs markedly from countries with binding quotas (Norway, France, Germany) or different board structures (Germany’s two-tier system)^2^.

The remainder of the paper proceeds as follows. Section 2 describes our data sources and the measures of interest. Section 3 outlines our empirical methodology and identification strategy. Section 4 presents results on pay gaps and explores mechanisms. Section 5 examines heterogeneity by firm size and board nationality. Section 6 concludes with policy implications.

## Data

This section presents the data sources, key variables, and how they are constructed. It also discusses sample selection, the unit of observation, potential limitations, and summary statistics.

### Data sources

We use firm-level administrative data from the UK starting in the 2017/18 fiscal year. Two data sources are combined: (i) the UK Government Equalities Office (GEO), and (ii) Financial Assets Made Easy (FAME)^3^.

#### GEO

GEO curates a dedicated website (https://gender-pay-gap.service.gov.uk/) where large employers are legally required to report their gender pay gap (GPG) measures annually since 2017^4^. The mandate applies to companies that (i) are registered in Great Britain and (ii) employ at least 250 people. They publish their GPG figures both on their own website and on the official GEO portal. Organisations that are part of a group must report individually. Large employers, i.e., firms with at least 250 employees, by April 5 (end of the financial year) must calculate their GPG as of that date. They publish the figures by the end of the financial year. Any company that delays its reporting submission is flagged on the GEO portal. This process is repeated annually for about 10,500 firms. Companies with fewer than 250 employees report their GPG on a voluntary basis. The definition of an employee follows the government guidelines^5^. Throughout the year, a company may submit its report multiple times; we only keep the latest submission.

#### FAME

FAME is a proprietary dataset maintained by Bureau van Dijk (now Moody’s). It is the UK version of the European Amadeus or Orbis dataset. FAME includes administrative data for the population of incorporated companies in the UK. FAME draws most of its information from the UK Companies House^6^ filings and includes financial accounts, officer registers, and records on directors and People with Significant Control. From FAME, we collect firm-level data from financial assets and director characteristics such as name, gender, age, and appointment date for companies that submit their GPG reports in the GEO platform.

#### Data access

We retrieve GEO data from the dedicated website https://gender-pay-gap.service.gov.uk/viewing/download for each year. We access FAME data via the institutionally licensed platform. To link these two, we use the unique company identifier (Company Registration Number).

### Measures of interest

#### Gender Pay Gap (GPG)

Following the government guidelines, companies calculate the gender gap in the median (or mean) hourly (ordinary) pay, relative to men’s pay, i.e., in an algebraic form:

1 $$\begin{aligned} \frac{w_m-w_f}{w_m} \cdot 100 \end{aligned}$$

where $w_m$ and $w_f$ are the average wages for male and female employees, excluding firm partners pay, respectively. Further, firms report the gender gap in the median (or mean) bonus pay relative to men’s bonus pay. Note that in mid-March 2020, due to COVID-19, the pay transparency mandate was temporarily suspended^7^.

##### Hourly ordinary vs. bonus pay

‘Pay’ excludes non-cash remuneration such as vouchers. These are classified under bonus pay. Salary sacrifice schemes, like childcare vouchers, count as ordinary pay. The classification also depends on timing. Payments during the Relevant Pay Period count as ordinary pay. Payments within the Relevant Bonus Period count as bonus pay.^8^

**Fig. 1:**
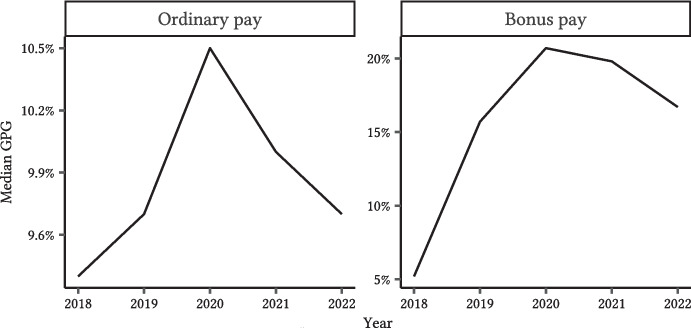
Raw company-reported GPG measures

Figure 1 shows the median raw GPG measures for the ordinary and bonus pay by year. Duchini et al. (2022) find that GPG narrowed by 18% after the transparency mandate. However, our data show that between 2018 and 2022, the GPG increased by 3.2 percentage points for ordinary pay and 11.5 points for bonus pay. Initially, bonus GPG was smaller than ordinary GPG. From 2019, they diverged significantly. Bonus GPG also has a much higher standard deviation, possibly due to firms reporting levels instead of percentage gaps. Due to these issues, we focus only on the GPG for ordinary pay in this paper.

#### Employee gender representation

We use two measures of employee representation by gender: the share of men at the top pay quartile and the female relative representation measure.

Companies are mandated to disclose in their report the proportion of male and female employees in each of their pay quartile, and the proportion of male and female employees who receive any bonus pay. While male and female shares are balanced at the bottom of the pay distribution, the top quartile is disproportionately male-dominated. This indicates that most firms employ more men in higher-paid roles. From these metrics, we use the *share of male employees at the top* pay quartile as an outcome to discuss the impact on the male over-representation at the upper part of the pay distribution.

**Table 1:** Ratio of companies that experience a female under-representation between two adjacent pay quartiles

Year	Q1/Q2	Q2/Q3	Q3/Q4
2018	61.90	65.38	69.03
2019	62.05	65.45	68.89
2020	65.61	68.41	69.43
2021	62.28	64.62	65.91

Note: The table shows the percentage (%) share of companies that report a female under-representation between two adjacent pay quartiles. Source: Authors’ elaboration based on https://gender-pay-gap.service.gov.uk

##### Relative representation

Further, based on the gender composition of the pay quartiles, we create a variable for *female relative representation* ($\text {RR}^f$). It captures the female worker representation between two adjacent pay quartiles across the pay distribution within each firm and is calculated as

2 $$\begin{aligned} \text {RR}^f = \frac{x_{i+1} - x_i}{x_i} \end{aligned}$$

where $x_{i}$ and $x_{i+1}$ are fractions of female employees between two adjacent pay quartiles. Between 2018-2021, 62% of companies have an underrepresentation of women in the lower middle pay quartile; this increases to 65% of companies in the upper middle pay quartile. In the top pay quartile, 69% of companies have an underrepresentation of women. Table 1 outlines these ratios by year. The under-representation is only relative to the adjacent quartile not the total fraction of the female workforce. If this were the case the extent of under-representation could be significantly higher. This measure is important as it indicates opportunities and promotion potentials for women. For example, if the upper middle pay quartile comprised 60% women, the top pay quartile would have more than a single-digit share.

The median under-representation of women across all pay quartiles and years is 7.3%. The distribution is right-skewed, meaning that the bulk of companies report a female under-representation, but only a few companies have a large over-representation.

#### Directors

##### Board of directors

FAME collects information on all directors, including board size. It does not distinguish well between executive and non-executive directors, but this does not matter much for our analysis. This is because the UK has a one-tier (unitary) board structure, wherein executive and non-executive directors jointly sit on a single corporate board. This contrasts with the two-tier system prevalent in some continental European countries, which separates management and supervisory functions across distinct boards. The unitary board model in the UK aims to balance strategic oversight with managerial accountability. Non-executive directors are tasked with monitoring executive actions and contributing to decision-making (Hopt and Leyens 2023; Financial Reporting and Council 2018).

##### Gender of directors

FAME offers the gender for each director. However, this is a variable derived by the data curator and is not publicly available from Companies House or submitted by the company. If the gender is not available in FAME, we use the genderizeR package (Wais 2006) in R to impute the gender based on the director’s first name. The algorithm identifies 2,288 different names accounting for different spelling in some cases^9^. Table 2 presents the directors descriptive statistics after imputing the director’s gender if not available in FAME. The average boardroom has around 6 women and 20 men directors, while less than 1 external company acts as directors (non-individual director).

**Table 2:** Directors, summary statistics

	Counts among boardrooms							Number of directors
Gender	min	q1	median	mean	sd	q3	max	
Female	0	1	3	5.63	7.62	7	195	55,006
Male	0	8	16	19.95	17.07	27	297	195,040
N/A	0	0	0	0.12	0.50	0	21	1,307
Non-individual director	0	0	0	0.67	1.25	1	31	7,152

Note: If the director’s gender is missing, ‘genderizeR‘ package imputes it based on their first name. Source: Authors’ elaboration based on FAME and genderize.io

##### Calculate the share

To calculate the share of female directors for a company, we include directors whose appointment date precedes the GPG report submission deadline. Using calendar year instead of fiscal year does not change our results.

The *share of female directors* calculates how many female directors are currently appointed among all current directors in the company *i* in year *t*, i.e.,

3 $$\begin{aligned} \text {share of female directors}_{i,t} = \frac{n^f_{i,t}}{n^m_{i,t}+n^f_{i,t}} \end{aligned}$$

where *n* stands for the number of directors; *m* and *f* refer to male and female, respectively. In a given year, a *current* female director is counted if her appointment date is before the due date of the GPG report.

##### Insider shareholders

Insider shareholders are the directors (or senior officials) who are shareholders - usually they own more than 10% of the voting shares (Jensen and Meckling 1976). FAME also collects information on shareholders, but only a small sample of our director population belongs to this category.

#### Average wages by gender

To calculate the within-firm average wage by gender, in the absence of employee pay microdata, we employ two assumptions:

1. the distribution of wages for men and women in each firm is log-normal^10^ - their joint distribution may not be.
2. the ranking of women (men) in the overall pay distribution within a firm comes from the knowledge of the proportion of women (men) in each pay quartile.

**Fig. 2:**
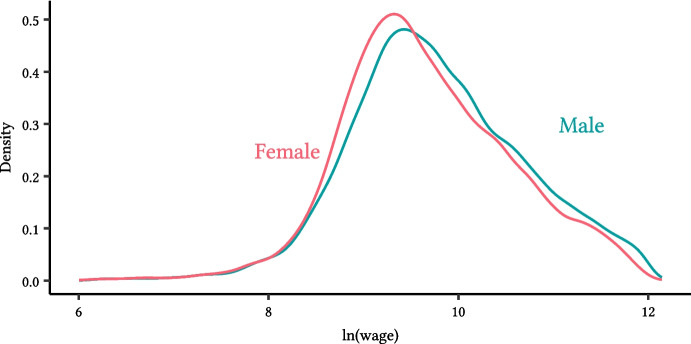
Wage distribution, by gender

Let $q_f$ as the quantile for women and $q_m$ and the quantile for men. Then we have

4 $$\begin{aligned} \ln (\text {Median difference of hourly pay between men and women}+1) = \sigma \cdot [\Phi ^{-1}(q_m)-\Phi ^{-1}(q_f)] \end{aligned}$$

where $\Phi ^{-1}$ is the inverse function of the standard normal distribution and $\sigma$ is the estimate of the standard deviation of wages within the firm. Once we obtain $\sigma$, we can calculate the natural logarithm of mean wages within the firm. To do so, we use the average wages and salaries per employee, as reported in FAME^11^. Finally, using the median difference of hourly pay between men and women, we find the average natural logarithm female and male wages. Figure 2 shows the resulting distribution of average wages for men and women across firms. Appendix C compares distributions from Fig. 2 to the ones received from the employee-employer matched dataset coming from the Annual Survey of Hours and Earnings (ASHE) for employers with at least 250 employees.

### Limitations

First, our primary outcome – the firm-level GPG – is company-reported. This means we do not observe individual worker wages or the full wage distribution within each firm. This reliance on an aggregate measure means we cannot control for employee composition differences within the firm (such as occupational mix or tenure) beyond what is implicit in the firm fixed effects. It also means the GPG is measured with some noise and potential reporting error. While the reporting process is standardized by the UK Government Equalities Office, there have been instances of mistakes (for example, some firms reportedly entered raw pay levels instead of percentages in the bonus gap field, leading to implausible values). We have taken care to use the median hourly gap (which is less prone to extreme misreporting than the bonus gap) and dropped obvious top and bottom 1% outliers. Any remaining reporting errors in the GPG would attenuate our estimates. Moreover, the pay gap data is limited to base pay: bonus payments and stock compensation are excluded from the reported median gap.

Second, an implication of the first point concerns the within-firm average wages we estimate. In the absence of individual wages at the firm level, we follow literature-motivated assumption. Real-world earnings distributions often display heavy tails or heteroskedasticity – particularly when men dominate high-paid leadership roles. This leads to skewness or differing dispersion across genders (Vattuone 2024). Calibrating log means from median pay gaps could thus understate true differences if outliers disproportionately affect one gender.

Third, the UK mandatory pay transparency covers only firms with 250 or more employees, by law. Thus, our sample is restricted to relatively large companies. The results may not generalize to small and medium enterprises, where board structures and dynamics differ (and where female leadership might have a different impact on pay practices).

Fourth, there was an interruption in the reporting in 2020: due to the COVID-19 pandemic, the government suspended mandatory GPG disclosures for the 2019/20 reporting year. While many firms still reported voluntarily, some did not, leading to a possible selection issue in the 2020 data. We include year fixed effects and controls to account for any aggregate shocks. The variation of our instrument is largely cross-sectional (regional), which accounts for the temporary reporting suspension. Nonetheless, the 2020 gap dip should be interpreted with caution, as it may partly reflect which firms chose to report (firms with better gender outcomes might have been more willing to continue reporting during the suspension). In our analysis, we have checked that excluding 2020 entirely does not materially change the estimates.

Fifth, even though FAME assigns gender to most of the directors’ names, it still has many with missing information. If a director’s gender in FAME is missing, we impute it using the R package genderizeR. The package assigns the gender based on the first name of each director. However, this depends on a dictionary of American names. Therefore, compound names or names that are not common between American and British English may be excluded. The way the package assigns gender to names is based on probabilities. We keep the highest probability for each distinct name.

### Sample and summary statistics

To link GEO and FAME data, we use the unique company identifier (“Company Registration Number”)^12^. Hence, the final dataset includes company information on GPG (i.e., median difference of hourly pay between men and women, share of women in each pay quartile), details about their financial status, and details of their directors.

In GEO data, 11,193 unique companies report their GPG measure between 2017/18 and 2020/21. FAME has information for 11,086 of them, which allows 99% matching. Among the companies in FAME, 329 (3.0%) become inactive. FAME collects information about 258,505 unique directors who are either individuals or companies (non-individuals). We restrict the sample to the companies whose directors are individuals and not third companies.

Further, we exclude companies that do not report their SIC code^13^, local authority, year or employer size^14^. In addition, we exclude companies from the public sector as the information in FAME is limited. The public sector companies are those whose:

1. 5-digit SIC code is equal to 1 or 84110^15^
2. GPG report is due by 31 March.

Finally, to alleviate concerns about outliers, we trim the top and bottom 1% of GPG distribution, share of current female directors and firm productivity. In this setting, we use company turnover per employee as a proxy for firm productivity. After all sample restrictions, we have 8,411 unique companies for which FAME has full information resulting in 26,677 company-year pairs.

Table 3 reports the summary statistics of the final sample.

**Table 3:** Summary statistics; no outliers

Variable	N	Mean	Std. Dev.	Min	Pctl. 25	Pctl. 50	Pctl. 75	Max	Source
Median % difference of hourly pay between men and women	26,677	12.27	16.17	-220.30	0.90	9.70	22.00	100.00	GEO
Firm age	26,677	30.85	23.56	0.11	14.12	24.88	39.32	120.80	FAME
% share of female employees	26,677	44.25	24.49	0.00	22.80	42.30	64.25	100.00	Calculated based on GEO and FAME
Share of current female directors	26,677	0.12	0.16	0.00	0.00	0.00	0.20	1.00	Calculated based on FAME
Turnover per employee (in £)	26,677	230,499.79	587,839.96	0.42	47,700.54	105,625.18	226,331.64	19,375,703.50	FAME
Number of employees	26,677	1,499.30	6,284.11	2.00	338.00	520.00	1,036.00	275,151.00	FAME
Liquidity ratio (x)	26,677	1.83	2.88	0.01	0.83	1.25	2.04	99.11	FAME
Wages & salaries GBP	26,677	53,232.67	381,431.04	8.11	9,450.00	16,389.00	35,225.00	51,100,000.00	FAME
Profit per employee (unit) GBP	26,677	13,452.22	88,836.49	-1,837,833.88	-342.37	2,980.92	12,381.68	1,918,196.43	FAME
log(Turnover per employee (in £))	26,677	11.62	1.11	-0.87	10.77	11.57	12.33	16.78	FAME
Size of employer	26,677								GEO
... 250 to 499	12,737	0.48							GEO
... 500 to 999	7,014	0.26							GEO
... 1000 to 4999	5,790	0.22							GEO
... 5000 to 19,999	943	0.04							GEO
... 20,000 or more	193	0.01							GEO
Return on total assets (%)	26,677	4.29	26.92	-894.43	-0.53	4.07	10.24	917.36	FAME
Number of current directors	26,677	5.96	3.85	1.00	3.00	5.00	8.00	74.00	FAME
Log average male wage	25,567	10.490	0.63	3.3900	10.13	10.51	10.87	14.24	Calculated based on GEO and FAME
Log average female wage	25,567	10.390	0.58	3.2900	10.08	10.40	10.70	14.00	Calculated based on GEO and FAME
Share of foreign directors	25,912	0.041	0.12	0	0	0	0	1	Calculated based on FAME

Source: Own elaboration based on FAME and gender-pay-gap.service.gov.uk

## Methodology

### Estimation strategy

The observation unit of this analysis is company $i$ in a given year $t$ in Local Authority District $k$ that operates in a 2-digit SIC $j$. Hence, the reduced form is:

5 $$\begin{aligned} y_{itkj} = \beta _0&+ \beta _1 \cdot \text {share of female directors}_{itkj} \nonumber \\&+ \beta _2 \cdot x_{itkj}+\gamma _i + \vartheta _t\cdot \eta _k\cdot \delta _j+v_{itkj} \end{aligned}$$

where *y* indicates the outcome of interest: GPG, share of men at the top of pay quartile, female relative representation, and within-firm average wage for men and women. *x* is a vector of controls: logarithm of turnover per employee, firm age, share of female employees, size of employer, profit per employee, liquidity ratio. *Turnover per employee* proxies for firm productivity, capturing output per worker while allowing comparisons across firms of different sizes and industries. $\beta _1$ captures the association between the increasing share of female directors and GPG.

In this exercise, we include company fixed-effects and the fixed-effects of local authority district, year and 2-digit SIC interacted. The interaction is necessary to control for differences across different LAD, year, and 2-digit SIC that are constant over time and are not captured by other control variables in the model. Standard errors are robust to heteroskedasticity and account for the clustering of firm observations within local authorities, time, and 2-digit SIC code.

### Identification strategy

Firms choose their board of directors, employees and production technology. Therefore, looking directly at the impact of female directors on a firm-level outcome, like the company-reported GPG, may raise some endogeneity-related concerns due to selection bias coming from non-random assignment of directors to firms. This endogeneity may be related to either unobservable or observable firm characteristics. In particular,

- **within-firm:** employee composition may be more female-weighted in female-directed companies than male-directed ones;
- **within-board:** male and female directors may systematically differ in terms of innate ability. This means that firms may systematically choose women for their boards, not due to their gender, but because of their higher ability;
- **between-firm:** a group of firms may be more productive than others due to unobservables. This unobserved heterogeneity may not be randomly assigned between firms directed by men and women. Firms with higher productivity may be more likely to have good corporate governance^16^ and a more diverse board of directors (reverse causality).

**Fig. 3:**
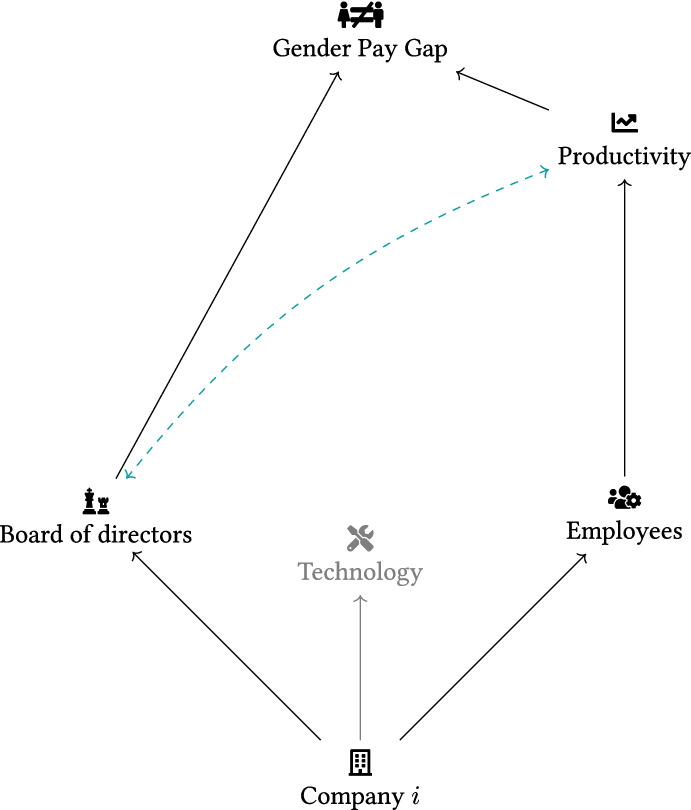
Illustration of identification. Source: Authors’ elaboration

Case (a) denotes heterogeneity in an observable firm characteristic, while cases (b) and (c) concern unobservables. Figure 3 illustrates the aforementioned relationships.

To address selection bias and endogeneity, we employ several strategies. First, we include time-varying controls for firm and employee characteristics. Second, we use firm fixed effects and interacted location-time-industry fixed effects to capture time-invariant heterogeneity, following the literature on within-firm variation in female management (Theodoropoulos et al. 2022; Flabbi et al. 2019; Gagliarducci and Paserman 2015; Albanesi and Olivetti 2009). However, since fixed effects cannot address time-varying unobservables, our main strategy employs a shift-share instrumental variable approach.

Further, the soft regulatory measures and voluntary targets may have played a significant role in driving gender diversity at the top of UK companies^17^. They induced a steady, economy-wide increase in female board participation rather than a sharp one-time shock. Because the rise in female directors was gradual and did not occur at the same time across firms, any general effects of these voluntary targets are captured by time trends in our data. We include year fixed effects in all our estimations to absorb broad economy-wide changes in board female representation over time.

### Bartik-type IV

Unobserved shifts in organizational practices (e.g., corporate governance) may both increase the likelihood of appointing female leadership and directly affect compensation structures (i.e., wages that affect the GPG). Such dynamics are not absorbed by firm fixed effects, which capture only time-invariant unobservables. To account for this, we construct a shift-share instrumental variable (IV) drawing on a Bartik (1991)-style approach, as used in Flabbi et al. (2019). In this type of IV, shocks and shares vary at different levels. Our IV interacts the initial firm-level indicator of female leadership with contemporaneous region-level trends in female leadership^18^. The identifying assumption is that regional changes in female leadership are correlated with firm-level leadership appointment choices (IV is relevant) but orthogonal to firm-specific time-varying shocks that may independently affect wages (IV is exogenous). Having constructed our IV, we follow a two-stage least square estimation and cluster standard errors at the company level.

In more detail, Bartik (1991) uses average industry growth rates at the national level to proxy latent labour demand shocks interacted with beginning-of-the-period industry employment shares at a local level. Here, we use the beginning-of-the-panel share of female directors at the firm level and interact it with the growth rate of the share of female directors at the regional level. Regions are 12 International Territorial Level (ITL)-1 areas, which are broader than the local authorities^19^. The trend at the regional level is correlated with the share of female directors of firms within a region; making our IV relevant. However, it is not correlated with any time-varying shocks that may endogenously affect wages and female directorships within a specific firm; making our IV exogenous. Appendix B.4 discusses the relevance and exclusion restrictions in more detail.

#### IV construction

The shift-share instrument is constructed using the following algorithm:

- **Step 1: base year** Set as the base year the first year the panel begins for each firm. This allows firms that enter later to have a different base year than already existing firms. This step assumes that the values of the share of female directors in the base year are exogenous given all controls.
- **Step 2: average by year and region across firms, excluding given firm** For each firm $i$ in region $r(i)$ in year $t$, calculate the average of the share of current female directors by region and year excluding firm $i$, i.e.,
  6 $$\begin{aligned} \overline{\text {share of female directors}_{-i, t,r(i)}} \end{aligned}$$
  Firm *i* is excluded to avoid any endogeneity coming from this firm affecting the average at the regional level. Appendix B.3 presents estimates when excluding both the 2-digit SIC sector and region of firm *i*.
- **Step 3: growth rate** Calculate the growth rate of the average from Eq. 6 by region and year relative to the base year:
  7 $$\begin{aligned} g_{i,t, r(i)} = \frac{\overline{\text {share of female directors}_{-i, t, r(i)}}}{\overline{\text {share of female directors}_{-i, \text {base year}, r(i)}}} \end{aligned}$$
- **Step 4: IV** Construct the IV ($z$) by interacting the base year value of the share of female directors ($\text {share of female directors}_{i, \text {base year}}$) with the growth rate calculated in Eq. 7
  8 $$\begin{aligned} \widetilde{z_{i,t}} = \text {share of female directors}_{i, \text {base year}} \cdot g_{i,t, r(i)} \end{aligned}$$

The “share,” namely the share of female directors$_{i, \text {base year}}$, is the share of female directors of a given company in the base year. The “shift-er,” i.e., $g_{i,t, r(i)}$, gives the proportion of the average share of female directors in a year *t* relative to the base year across all companies having excluded the given firm. We exclude the given company as it may affect the average share of current female directors in the ITL-1 region. Hence, $\widetilde{z_{i,t}}$ is the predicted value of the share of female directors for company *i* in year *t*, with the assumption that the distribution of female directors aligns with the observed distribution of female directors in the base year.

Goldsmith-Pinkham et al. (2020) show that the two-stage least squares using a Bartik estimator is numerically equivalent to the generalised method of moments (GMM) estimator that uses shares as instruments. Hence, the main identification source relies on the shares instead of the shifts. The share has predictive power for the growth rate of the share of current female directors, as some companies appoint more women on their boards, while others appoint fewer or none.

**Table 4:** Effects of female directors on GPG; 2SLS; second-stage estimates

	Dep. var.: Median difference of hourly pay between men and women/100							
	(1)	(2)	(3)	(4)	(5)	(6)	(7)	(8)
Share of current female directors (fitted)	-0.040***	-0.016	-0.007	-0.028**	-0.028**	-0.028**	-0.043***	-0.043***
	(0.014)	(0.014)	(0.012)	(0.012)	(0.012)	(0.012)	(0.012)	(0.012)
F-statistic for IV in first stage	50,359.1	48,803.4	28,723.8	27,173.9	27,161.8	27,161.2	26,701.4	26,699.2
N	26,677	26,677	26,677	26,677	26,677	26,677	26,677	26,677
R2 adj.	0.066	0.084	0.303	0.308	0.308	0.308	0.318	0.318
FE: Firm	No	No	Yes	Yes	Yes	Yes	Yes	Yes
FE: LAD $\times$ year $\times$ 2-digit SIC	No	No	Yes	Yes	Yes	Yes	Yes	Yes
FE: LAD	Yes	Yes	No	No	No	No	No	No
FE: Year	Yes	Yes	No	No	No	No	No	No

* p < 0.1; ** p < 0.05; *** p < 0.01

Note: Robust standard errors clustered at Local Authority level (models 1-2) or company level (models 3-8). The company-reported Median difference of hourly pay between men and women is our Gender Pay Gap (GPG) measure. Additional controls by specification: Logarithm of turnover per employee (models 2-8), firm age (models 3-8), Employer size (models 4-8), Share of female employees (models 4-8), Profit per employee (models 5-8), Liquidity ratio (model 6-8), Logarithm of number of current directors (board size; models 7-8), Return on total assets (in percentage; model 8). For brevity, we report only the coefficients of interest. Table B.5 reports the first stage estimates. Table B.1 reports the reduced-form fixed effects specifications

## Results and discussion

### Effect on the company-reported gender pay gap

Table 4 presents the impact of board gender composition on the company-reported GPG. It reports the second-stage results for each specification. Each specification includes progressively additional controls. Specification 1 does not include any control and has only fixed effects. Additional controls by specification are: logarithm of turnover per employee (models 2-8) as a proxy for the firm labour productivity, firm age (models 3-8), employer size (models 4-8), share of female employees (models 4-8), Profit per employee (models 5-8), Liquidity ratio (model 6-8), the board size (models 7-8), and Return on total assets (model 8). The “share of female directors (fitted)” comes from the first stage, and it is the fitted values when the share of female directors is regressed on $\widetilde{z}$ (Eq. 8). Appendix B.4 shows the first-stage estimates and discusses the instrument relevance and exogeneity conditions. The first-stage specifications do not show any weak instrument issues. The first-stage regressions report a positive and statistically significant coefficient for the instrument, supporting its relevance argument. However, this matters little for identification purposes. In fact, for identification, the sufficient condition is the correlation between the instrument and the share of female directors, but not any time-variant firm-specific heterogeneity. First-stage specifications include the same controls as their corresponding second-stage specifications.

Across all model specifications, the coefficient on the female director share is negative and statistically significant, indicating that more women on the board are associated with a lower company-reported pay gap. In the fullest specification (Table 4), a 1 percentage point increase in the share of current female directors decreases the company-reported GPG by 0.043 percentage points.

To economically contextualise this result, consider the following. The median GPG in our sample is 9.70% (Table 3), which means that if a man earns £1, his female counterpart earns 90.3p. A 1p.p. increase in the share of current female directors will translate into female earnings from 90.30p to 90.343p per £1 of male earnings. This is an improvement of around 0.86p if men earn £20/hour. As a result, this is a 0.05% increase in female earnings. For a woman earning annually £50,000 when the GPG is 9.7%, this 1 percentage point increase in female directors would translate to roughly an additional £23.80 per annum. Scaling this coefficient, moving from 12% to 50% female directors in a boardroom (a 38p.p. increase) would lower the gap by 1.63 pp—about one-sixth of the sample’s 9.7-pp median gap. Scaled to comparable changes in representation, our estimated impact of female board share on firms’ median GPG (1.0–1.4 pp for a 33-pp increase) is similar to the 1.2-pp reduction reported by Sondergeld and Wrohlich (2023) for female top management in Germany.

For robustness, appendix B.3 replicates this same Bartik-IV approach excluding not only firm *i*, but also its corresponding 2-digit SIC sector when calculating the average of the share of female directors by year and region. Results remain significant and the effect persists in the same direction. Further, we estimate the reduced-form using fixed effects. The results have the same direction, but their magnitude is smaller for the coefficient of interest.

**Table 5:** Effects of female directors on male and female wages

	Dep. var.: Log of within-firm average wages for			
	men		women	
	IV	FE	IV	FE
	(1)	(2)	(3)	(4)
Share of current female directors	0.073*	0.085***	0.113***	0.119***
	(0.039)	(0.029)	(0.036)	(0.028)
F-statistic for IV in first stage	25,743.5		25,743.5	
N	25567	25,567	25,567	25,567
R2 adj.	0.532	0.532	0.519	0.519
FE: Firm	Yes	Yes	Yes	Yes
FE: LAD $\times$ year $\times$ 2-digit SIC	Yes	Yes	Yes	Yes

*p < 0.1; ** p < 0.05; *** p < 0.01

Note: Robust standard errors clustered at Company level. Additional controls: logarithm of turnover per employee, firm age, Employer size, Share of female employees, Profit per employee, Liquidity ratio, Logarithm of number of current directors (board size), Return on total assets (in percentage). For brevity, we report only the coefficient of interest. IV refers to our Bartik-type IV and reports estimates from the second-stage fitted values. Both models 1 and 3 share the same first-stage equation and estimates. FE refers to a fixed effects model (Eq. 5).

### Mechanisms: channels of influence

While the overall impact on the company-reported GPG is quantitatively small, female board representation appears to influence several underlying aspects of firm pay structure. This is consistent with women directors having positive spillover effects on female employee outcomes. We explore three key channels through which female directors can narrow within-firm gender disparities.

#### Differential wage effects

Although firms disclose their GPG rather than individual wages, we estimate within-firm average wages separately for men and women. We evaluate the effect of board gender composition on these outcomes (Table 5). The number of observations decreases because FAME does not include information on the wages and salaries in certain years and/or firms. Using the IV estimates, a 1 p.p. increase in the share of female directors yields a within-firm average rise in female wages of about 0.11%, while male wages rise by 0.07%. A move from 0% to 50% female directors would correspond to roughly 3.7% higher wages for men and 5.7% for women. Looking at the magnitude, the effect is significantly larger for female wages than for male wages (and their difference is statistically significant).

Although these percentage changes are small, the gap in these effects (0.04% extra gain for women per pp) accumulates to lower pay inequality over time. This differential wage impact directly contributes to narrowing the GPG and is consistent with the idea that female leaders in decision-making positions work to reduce discriminatory pay practices (Flabbi et al. 2019; Blau and Kahn 2017). In practical terms, women directors may advocate for pay raises, adjustments, or salary benchmarks that particularly benefit women employees (who may have been underpaid relative to men), thereby lifting women’s wages closer to parity. Several studies find similar gender-specific effects of female leadership on wages (Bell 2005 for pay premiums; Matsa and Miller 2013; Melero 2011 for working environment; Huber et al. 2022 for fairness in compensation). Our findings reinforce that dynamic: gender-diverse boards tend to boost women’s earnings more than men’s, directly reducing wage inequality within the firm.

#### Representation across the pay distribution

Table 6 looks at two additional outcomes: the share of male employees at the top pay quartile (columns 1-2), and the female relative representation across the pay distribution (columns 3-4). Estimates show that female directors are associated with a decrease in male over-representation in the top pay quartile. Moreover, there are female representation gains that are not confined to the top. The female relative presence improves across all pay quartiles under more gender-diverse boards. Female directors may help create or reinforce opportunities for women at multiple levels within organisations. That can be through influencing promotions, mentoring, or advocating for equitable talent development. This result contributes to the spillover literature by showing both vertical and horizontal spillovers Von Essen and Smith (2024); Kunze and Miller (2017); Matsa and Miller (2011). Women at the top improve the position of women below both by helping more of them reach high-paying positions (vertical spillover) and by generally fostering an environment where women are more present across the pay distribution (horizontal spillover). Our findings align with Kirsch (2022), who documents that female directors engage in active ‘equality-related actions’ rather than serving as passive role models.

**Table 6:** Effects of female directors on employee representation across the pay distribution

	Dep. var.: Share of men in top quartile		Dep. var.: Female relative representation	
	IV	FE	IV	FE
	(1)	(2)	(3)	(4)
Share of current female directors	–0.048***	-0.041***	0.575***	0.209***
	(0.007)	(0.005)	(0.159)	(0.062)
F-statistic for IV in first stage	26,699.2		26,699.2	
N	26677	26677	26677	26677
R2 adj.	0.911	0.911	-0.104	–0.101
FE: Firm	Yes	Yes	Yes	Yes
FE: LAD x year x 2-digit SIC	Yes	Yes	Yes	Yes

* p < 0.1, ** p < 0.05, *** p < 0.01

Note: Robust s.e. clustered at Company level. Additional controls: logarithm of turnover per employee, firm age, Employer size, Share of female employees, Profit per employee, Liquidity ratio, Logarithm of number of current directors (board size), Return on total assets (in percentage). For brevity, we report only the coefficients of interest. IV refers to our Bartik-type IV and reports estimates from the second-stage fitted values. Both models 1 and 3 share the same first-stage equation and estimates. FE refers to estimates from a fixed effects model (Eq. 5)

#### Performance-related pay allocation

Female directors can better recognise the effort of their female employees and give them the opportunity to access performance-related payments (PRP; e.g., end-of-year bonus) (Theodoropoulos et al. 2022; Melanie and Kaya 2022). PRP offer directors the flexibility to shape pay outcomes among employees. We show evidence that boards with at least one female director tend to ensure women are not overlooked in performance pay decisions. We show how the share of women (men) who receive a PRP changes over time in the presence of female directors. This step does not test whether female directors design pay menus. Instead, it tests if board gender composition can determine who receives a pay reward^20^.

**Fig. 4:**
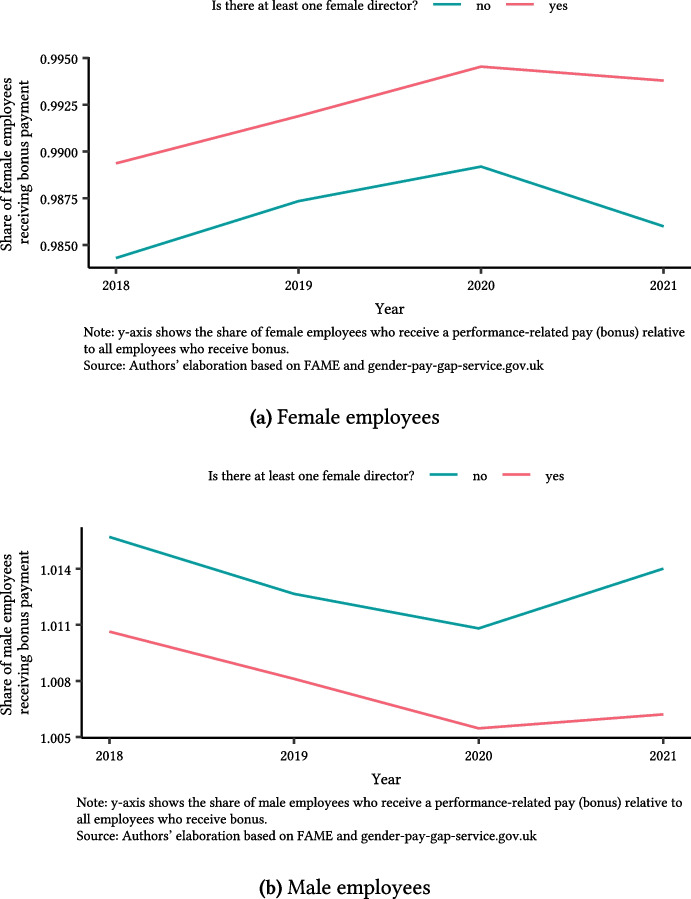
Performance-related payments and female directors

Figure 4 shows that male employees receive PRP more often than women regardless of board composition, but female directors are consistently associated with improved PRP access for women (panel a) though not for men (panel b).

One interpretation is that female directors may be more attuned to the contributions of women and thus advocate for recognizing those contributions financially. Prior studies suggest female managers often implement fairer evaluation and compensation practices. For example, Theodoropoulos et al. (2022) find that female supervisors are better at identifying and rewarding productive effort by women. This has important implications because bonus pay can be a significant part of earnings, especially in high-level positions. Ensuring women receive their fair share of such pay helps reduce overall earnings inequality. Our primary GPG measure does not include bonuses, so this channel operates beyond our main outcome. Yet, it points to real gains for women that a narrow wage statistic might miss.

### Theoretical perspectives and interpretation

We interpret the small, but robust, reduction in the company-reported GPG associated with higher female board representation through three complementary lenses: Agency Theory, Resource Dependence Theory, and Social Role/Role Congruity Theory. Together, these frameworks explain why the effect exists, why it is modest in magnitude, and why it is stronger in mid-sized and large firms.

#### Agency theory (monitoring and the “discrimination cost”)

In classic agency models, boards mitigate managerial agency problems through monitoring and incentive alignment Jensen and Meckling (1976). The literature shows that gender-diverse boards intensify monitoring (e.g., higher meeting attendance and greater committee participation), thereby improving oversight of policies that can embed bias (Adams and Ferreira 2009). If discrimination is an inefficiency (“agency cost”) that wastes talent and risks legal/reputational penalties, stronger oversight should push toward more merit-based pay and advancement. This mechanism is consistent with our within-firm patterns: slightly faster wage growth for women than men, narrower male dominance at the top pay quartile, and smaller gender gaps in bonus incidence. In this view, adding female directors helps the board detect and correct biased pay-setting practices and promotion bottlenecks, yielding small but systematic improvements in pay equity.

#### Resource dependence theory (board capital, and HR practices)

Resource Dependence Theory (RDT) views boards as providers of “board capital”, i.e., expertise, networks, information channels, stakeholder access, and legitimacy (Hillman et al. 2007; Hillman and Dalziel 2003). Increasing female representation broadens this capital, strengthening links to external stakeholders (customers, investors, regulators) who increasingly value diversity and fair employment practices. External stakeholder pressure can diffuse internally into HR systems. Hence, HR practices form clearer pay frameworks, fairer performance evaluation, and broader talent pipelines—which helps explain our horizontal spillovers (more even female representation across pay quartiles) as well as improvements in bonus access. RDT also predicts heterogeneity: larger firms face greater external scrutiny and have more formalized systems that can translate board guidance into organization-wide practice. This maps onto our finding that effects are more pronounced in mid-sized to large firms (250 - 5,000 employees) in the coming section.

#### Social role theory (norms, role modelling, and critical mass)

Social Role and Role Congruity theories explain how gendered expectations shape evaluations of leaders and the adoption of inclusive practices (Eagly and Karau 2002). Because leadership roles are stereotyped as “agentic”, women often face higher barriers to influence. When present in sufficient numbers, however, female directors can both shift norms (role modelling, signalling the value of female contributions) and advocate for practices that foster equitable pay and advancement. This helps reconcile our vertical spillovers (more women reaching the top quartile) with the horizontal diffusion across the distribution: visibility at the top changes perceptions and strengthens sponsorship throughout the organization. While a “queen bee” syndrome dynamic can arise in male-dominated settings (Srivastava and Sherman 2015; Bednar and Gicheva 2014), our estimates point to net positive spillovers in the UK one-tier board context and under pay-transparency pressure. Therefore, our results are in line with recent evidence on leadership spillovers (e.g., Maida and Weber 2022; Von Essen and Smith 2024).

#### Summing up these theories

Together, these theories suggest that gender-diverse boards: (i) tighten oversight of potentially biased processes (agency theory), (ii) import resources and legitimacy that sustain inclusive HR systems (RDT), and (iii) shift norms that aid both vertical advancement and horizontal representation (social role theory).

They also explain why the effect size is modest in percentage-point terms: boards influence pay structures indirectly, through governance and systems, rather than setting individual wages. They rationalize heterogeneity: where systems are formalized and stakes are high (e.g., in large firms), board influence more readily translates into measurable changes in pay dispersion and rewards. Overall, our findings complement prior work showing that female leadership can narrow gender gaps, while clarifying that board-level representation produces detectable but incremental improvements in pay equity in the UK.

## Heterogeneity

### Heterogeneity by employer size

The pay-gap reduction associated with the female presence in corporate boardrooms varies across firm types (Melanie and Kaya 2023). For example, in appendix B.2, when we restrict the sample to mid-sized and large firms (those with 250–5,000 employees), the coefficient of interest becomes more negative (around –0.05 in some specifications, vs. –0.04 for the full sample), indicating a larger impact of board gender diversity on the GPG. In contrast, in an analysis focusing on the extra-large companies (those with over 5,000 employees), the effect is muted and not always statistically significant. This pattern suggests diminishing returns to board diversity in the absolute largest firms, possibly because these organizations are more complex or have formalized pay structures that leave less scope for board influence.

Our finding that female directors significantly reduce pay gaps in mid-sized and large firms (250-5,000 employees), but not in extra-large firms (5,000+), stands in contrast to some prior studies examining gender gaps under female leadership. For instance, Kritikos et al. (2024) study the gender of firm owners in Finland and report that while female-owned firms have a 2–3 percentage-point lower pay gap than male-owned firms on average, this advantage disappears in large firms (50+ employees).

Our results suggest that board-level gender diversity may exert a more persistent and sizable influence on gender pay differences across a range of firm sizes. Corporate boards set the tone and policies that shape pay structures, whereas female CEOs or owners in large firms might be constrained by existing organizational frameworks. Unlike female entrepreneurs studied by Kritikos et al. (2024), female directors in the UK one-tier board system can use their oversight role to push for fairer compensation practices throughout the firm. Our UK sample indicates that greater gender balance at the top is associated with measurably fairer pay outcomes. This new evidence complements prior research by highlighting that the “trickle-down” effects of women in leadership may extend to the boardroom level, not just owners. These effects vanish in extra-large firms but not in mid-sized and large firms (at least in our institutional context). This contrasts with earlier findings and thus contributes a novel insight: female representation in governance matters most in moderately complex organizations (250-5,000 employees), where boards retain sufficient influence over pay structures.

### Heterogeneity by board nationality composition

Do female directors have different effects on GPG depending on board nationality composition? For this analysis, a a board is classified as non-UK if 51% of members come from any country other than the UK^21^. Ahamed et al. (2019) use the share of foreign nationality directors and finds that additional foreign-nationality directors decrease GPG. However, Ahamed et al. (2019) do not consider that both types of firms may differ in unobservables. We address this potential bias by conducting separate estimations for each type of company.

**Table 7:** Effects of female directors on GPG and wages, by board nationality

	Dep. var.: Median difference of hourly pay between men and women				Dep. Var.: ln(average male wages)				Dep. Var.: ln(average female wages)			
	UK-nationals^a^		non-UK-nationals^b^		UK-nationals^a^		non-UK-nationals^b^		UK-nationals^a^		non-UK-nationals^b^	
	IV	FE	IV	FE	IV	FE	IV	FE	IV	FE	IV	FE
	(1)	(2)	(3)	(4)	(5)	(6)	(7)	(8)	(9)	(10)	(11)	(12)
Share of current female directors	–0.043***	-0.033***	0.005	-0.008	0.059	0.074**	0.336	0.183	0.103***	0.110***	0.326	0.191
	(0.012)	(0.009)	(0.062)	(0.044)	(0.039)	(0.030)	(0.267)	(0.152)	(0.037)	(0.028)	(0.262)	(0.147)
F-statistic for IV in first stage	24,897.2		669.6		24,452.3		662.5		24,452.3		4.5587	
N	24818	24818	1094	1094	24422	24422	1080	1080	24422	24422	1080	1080
R2 adj.	0.331	0.331	0.089	0.089	0.534	0.534	0.289	0.291	0.520	0.521	0.337	0.241
FE: Firm	Yes	Yes	Yes	Yes	Yes	Yes	Yes	Yes	Yes	Yes	Yes	Yes
FE: LAD $\times$ year $\times$ 2-digit SIC	Yes	Yes	Yes	Yes	Yes	Yes	Yes	Yes	Yes	Yes	Yes	Yes

* p < 0.1; ** p < 0.05; *** p < 0.01

^a^ A board of directors is owned, or managed, by UK nationals if 51% of the board members come from the UK. If a director holds dual nationality and one of them is British nationality, they are classified as UK nationals.

^b^ A board of directors is owned, or managed, by non-UK nationals if 51% of the board members come from any country other than the UK.

Note: Robust standard errors clustered at the Company level. Additional controls for all specifications: Logarithm of turnover per employee, Firm age, Employer size, Share of female employees, Profit per employee, Liquidity ratio, Logarithm of number of current directors (board size), Return on total assets (in percentage). For brevity, we report only the coefficients of interest. IV refers to our Bartik-type IV and reports estimates from the second-stage fitted values. Both models 1 and 3 share the same first-stage equation and estimates. FE refers to a fixed effects model (Eq. 5)

Table 7 replicates the estimation strategy followed earlier in the paper for two distinct subsamples: those with boards predominantly consisting of UK nationals and those with boards predominantly consisting of non-UK nationals. It reveals two important results. First, we note that both groups of companies present similar distributions in terms of their observables (e.g., turnover per employee). Second, boards predominantly comprised of UK nationals exhibit a negative relationship with the GPG and a positive association with female wages. Conversely, boards with a majority of directors from non-UK countries do not demonstrate any significant impact on the examined outcomes when the gender composition changes. Consequently, when considering jointly the nationality and gender composition of boards, female directors only reduce GPG in boards where more than 51% of directors are UK nationals. This suggests that local knowledge of the UK institutional and cultural environment is necessary for directors to effectively influence internal pay practices.

Evidence on “local embeddedness” shows that leaders with strong local ties deliver measurably stronger CSR and stakeholder outcomes—mechanisms closely related to wage fairness and equality initiatives (Chang et al. 2025). By contrast, foreign-led or highly international boards can face monitoring and coordination frictions that blunt their influence on sensitive domestic outcomes like executive pay discipline and compliance, consistent with evidence that firms with foreign independent directors show weaker oversight at home (e.g., higher CEO pay, more misreporting, lower performance) (Masulis et al. 2012).

## Conclusion

This paper exploits rich firm-level administrative data for employers with at least 250 employees between 2017 and 2021. It provides evidence that female directors reduce company-reported gender pay gaps, though the economic magnitude is modest.

Using a Bartik-style instrumental variable approach that exploits regional variation in female board representation, we find that a one percentage point increase in female directors reduces the GPG by 0.043 percentage points or increases female earnings by 0.05%. This implies that even increases in board diversity to reach gender parity would reduce the gap by 1.6 percentage points, closing about one-sixth of the 9.7% median pay gap in our sample.

The effect operates through three mechanisms: (i) asymmetric wage increases favouring women (0.11% vs. 0.07% for men), (ii) improved female representation across all pay quartiles (not just the top), and (iii) more equitable allocation of performance-related pay. Effects are concentrated in mid-sized and large firms (250-5,000 employees) and are only present when UK nationals comprise over 51% of directors, suggesting local knowledge matters for implementing equity-enhancing practices. Our findings indicate that while board diversity generates positive spillovers consistent with agency and resource dependence theories, it must be complemented by broader organizational reforms to meaningfully address pay disparities.

### Policy implications

This paper contributes to the literature that looks at the mechanisms to decrease GPG beyond individual and workplace characteristics. Our results suggest two policy tools for reducing GPG: increasing female representation throughout organizations and appointing more female directors, in line with Theodoropoulos et al. (2022); Sondergeld and Wrohlich (2023) and Kunze and Miller (2017). These policy tools are more important in male-segregated sectors (Folke and Rickne 2022). This paper demonstrates that who sits in the boardroom matters for those on the shop floor, even if the effect is more evolutionary than revolutionary. As firms face increasing pressure from investors, regulators, and employees to address gender pay gaps, our evidence provides a realistic baseline for what board diversity can achieve. Female directors can be considered a positive work-culture element toward equal opportunities and treatment that shifts norms and affects preferences (Cullen and Perez-Truglia 2023; Chevalier 2007).

## Supplementary Information

Below is the link to the electronic supplementary material.

Supplementary file 1 (pdf 205 KB)

## Data Availability

This study uses three data sources with varying access conditions. First, the Government Equalities Office gender pay gap reporting data are publicly available at https://gender-pay-gap.service.gov.uk/. Second, FAME (Financial Analysis Made Easy) company accounts and directors’ data were accessed through the institutional licence held by King’s College London via the Bureau van Dijk/Moody’s platform; access is available to researchers at subscribing institutions. Third, the Annual Survey of Hours and Earnings (ASHE) is held by the Office for National Statistics and was accessed via the ONS Secure Research Service (SRS). Approved researchers may apply for access to ASHE microdata through the SRS. The code used for compiling the final dataframes and analysis is available on GitHub at https://github.com/ygalanak/GalanakisGosling2026.

## Footnotes

1 According to Business Population Estimates (BEIS 2022, table 25), large companies constitute 0.1% of the total company population, but employ 40% of the workforce and generate 49% of the total turnover. See (Fig. A.1).

2 Unlike these countries, the UK has pursued soft regulatory measures: the Davies Review in 2011 set voluntary targets of 25% female board representation for FTSE 100 companies by 2015, subsequently raised to 33% by the Review (2016), and 40% by the FTSE Women Leaders (2022). These initiatives lacked legal enforcement. They induced a steady, economy-wide increase in female board participation rather than a sharp one-time shock. Because the rise in female directors was gradual and pervasive across firms, any general effects of these voluntary targets are captured by time trends in our data. We include year fixed effects (and other controls) in all our models, which absorb broad economy-wide changes in board gender diversity over time.

3 The GEO data were last accessed on 22 October 2022. While updates may occur daily, they do not affect our results. Our analysis ends in 2021, as FAME contained complete data on turnover, employment, and directors only up to that year.

4 In 2015, when the government initiated discussions around pay transparency in the UK, women held 34% of managerial, director, and senior official roles (Government Equalities Office 2015), and just 23% of board positions in FTSE 100 companies (Vinnicombe and Tessaro 2022). Two years later, following consultation with employers, the government introduced the *Equality Act 2010 (Gender Pay Gap Information) Regulations 2017*.

5 Agency workers are included, but firm partners are excluded. Part-time and full-time staff are weighted equally.

6 Companies House is the UK’s registrar of companies. It is a government agency (sitting under the UK Department for Business and Trade) that maintains a public register of all limited companies and limited liability partnerships (LLPs) registered in the UK.

7 After 24 March 2020, firms were no longer required to report their GPG measures due to the pandemic. Some organisations, though, continued to publish their GPG indicators until October 2021. However, the number of observations for this year is significantly smaller than the previous two and the coming waves. Jones et al. (2022) find that companies that kept reporting, relative to non-reporters during the partial suspension, have 6% lower GPG a year after the suspension.

8 Example: If a firm’s snapshot date is 5 April 2018, and an employee earns £10,000 in ordinary pay and a £7,000 bonus during April, the full amount counts toward the GPG. For private and third sector firms, the Bonus Period is 6 April–5 April of the same year. For public sector firms, it is 1 April–31 March.

9 Compound names, or names including only the initial letter have not been identified.

10 The sample is comprised of companies with at least 250 employees. Therefore, this can be a reasonable assumption for large employers. The log-normality assumption is not unprecedented in the literature. Bílková (2019) analyses Czech wage data by firm size and models the entire wage distribution in each size category with a parametric log-normal curve. Vattuone (2024) develops an equilibrium on-the-job search model with matched employer–employee data; she assumes that wages are log-normally distributed, with the mean and variance potentially differing by worker type and firm class (employer group). Gender wage gap studies have also used log-normal wage offers (Braun and Figueiredo 2025; Kai 2016).

11 Alternatively, we have used the average remuneration per employee within a company. Remuneration is calculated in FAME as the sum of (a) wages and salaries; (b) social security costs; (c) pension costs and (d) other staff costs. This yields a slightly different distribution of wages.

12 This identifier is obtained by the UK Companies House upon registration.

13 If the SIC code is missing in a given year, the value reported for the previous year is used. If not available in GEO data, the value is imputed from FAME. If the company reports multiple SIC codes, we only use the main (or primary) sector.

14 In cases when the employer size is not provided in GEO data, the total number of employees in the respective year from FAME is used. This imputation step regards 746 observations. Companies with fewer than 250 employees report their GPG voluntarily. We exclude them from the sample and strictly keep those employers with at least 250 employees.

15 This category includes the following public-sector activities: Central government administration; Taxation schemes; Tax violation investigation services; Statistical services (public sector); Public fund services administration; Public debt services administration; Local government administration; General public administration activities; General personnel administration and operational services (public sector); Fundamental research administration (public sector); Fiscal services (public sector); Financial services (public sector); Executive and legislative administration (public sector); Economic and social planning administration (public sector); Duty and tax collection; Customs administration; Trust territory programme administration (public sector).

16 This means the relationship between firm productivity and corporate governance can be bidirectional; good corporate governance can increase productivity, but higher productivity can enhance good corporate governance.

17 The four key voluntary initiatives are: the Davies Review (2011–2015, (https://www.gov.uk/government/publications/women-on-boards-5-year-summary-davies-review); Hampton-Alexander Review (2016–2021, https://www.gov.uk/government/publications/ftse-women-leaders-hampton-alexander-review); FRC Corporate Governance Code (https://www.frc.org.uk/library/standards-codes-policy/corporate-governance/corporate-governance-code-guidance/); and, FTSE Women Leaders Review (FTSE Women Leaders 2022).

18 We choose to aggregate at the regional level and not at the sector level. Aggregating at the sector level may be endogenous to the wage setting at the firm level. Therefore, in this way, we satisfy the exogeneity condition for the wage determination.

19 Regions are: London, South East, South West, West Midlands, East Midlands, North West, North East, East of England, Yorkshire and The Humber, Scotland, Northern Ireland and Wales. For allocation of local authorities to regions, see here.

20 Alternatively, we could use the GPG measure in bonus to test the size of the rewards given to different groups of workers based on their gender. However, this may be biased in this data. Given that the GPG in bonus has a big standard deviation, which implies that some companies mistakenly reported the level and not the percentage gap between the male and female bonus. Further, we would need information on the average bonuses paid by each firm - this is not provided in FAME.

21 In case a board member holds a dual nationality, one of which is British, this member is classified as a UK national. Further, it is important to acknowledge the data limitation of our study in determining the nationality status of directors at the time of appointment, especially in cases where individuals may have acquired UK nationality after their initial appointment.
