# Supplementary material for: Mind the (gender pay) gap: the role of board gender composition
Source: J Popul Econ. 2026 Feb 21;39(1):16. doi: 10.1007/s00148-026-01159-x (PMC12924850; doi:10.1007/s00148-026-01159-x)
Supplement: Supplementary file 1 — (pdf 205 KB) [file 148_2026_1159_MOESM1_ESM.pdf]

A Cumulative share of workers

In this section, we look at the private sector employers with at least 250 employees from the GEO data. We plot the cumulative percentage of men and women against their median percentage difference of hourly pay. To do so, we exploit the information regarding the share of women (men) in each quartile of the pay distribution. Assuming that the average of all quartiles yields the share of women (men) in a company, we derive the number of female (male) workers. From FAME, we retrieve the annual number of company workers. Multiplying the number of workers by the share of female (male) workers in each company, we calculate the number of women (men). Figure A.1 reports that between 2018-2021, 9 out of 10 women work in a company that pays them less than men. 50% of women work in a company that pays them 10.5% less than men.

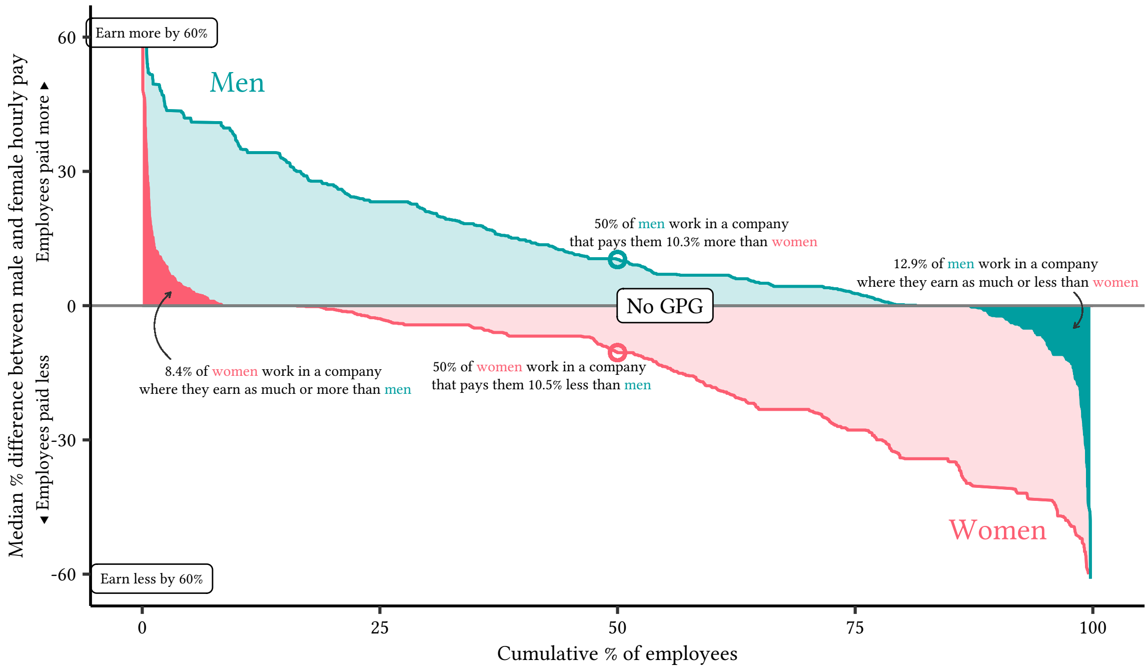

Note: Calculation average for 2017/18–2020/21.
y-axis is restricted between -60 and 60% - remaining values are available upon request.
Source: Own elaboration based on FAME and gender-pay-gap.service.gov.uk

Figure A.1: Cumulative share of workers by GPG; 2018-2021

B Robustness checks

Here we include a battery of robustness checks. First, we present the reduced-form regressions using fixed effects for our main outcome of interest, namely the company-reported GPG. Second, we run the same fixed effects model on three subsamples. Third, we show that the findings are unlikely to be driven by the GPG-reporting policy mandate. Fourth, we present an alternative methodological approach that uses the Bartik-type instrument which excludes both firm i and its corresponding 2-digit SIC sector.

B.1 Effect on the gender pay gap, fixed effects

Table B.1: Effects of female directors on GPG; fixed effects

	Dep. var.: Median difference of hourly pay between men and women/100							
	(1)	(2)	(3)	(4)	(5)	(6)	(7)	(8)
Share of current female directors	-0.024^{**}	-0.000	0.005	-0.013	-0.013	-0.013	-0.030^{***}	-0.030^{***}
	(0.012)	(0.012)	(0.009)	(0.009)	(0.009)	(0.009)	(0.009)	(0.009)
N	26,677	26,677	26,677	26,677	26,677	26,677	26,677	26,677
R2 adj.	0.066	0.084	0.303	0.308	0.308	0.309	0.318	0.318
FE: Firm	No	No	Yes	Yes	Yes	Yes	Yes	Yes
FE: LAD x year x 2-digit SIC	No	No	Yes	Yes	Yes	Yes	Yes	Yes
FE: LAD	Yes	Yes	No	No	No	No	No	No
FE: Year	Yes	Yes	No	No	No	No	No	No

* $p < 0.1$, ** $p < 0.05$, *** $p < 0.01$

Note: Robust s.e. clustered at Local Authority level (models 1-2) or company level (models 3-8). The company-reported Median difference of hourly pay between men and women is our Gender Pay Gap (GPG) measure. Additional controls by specification: Logarithm of turnover per employee (models 2-8), firm age (models 3-8), Employer size (models 4-8), Share of female employees (models 4-8), Profit per employee (models 5-8), Liquidity ratio (model 6-8), Logarithm of number of current directors (board size; models 7-8), Return on total assets (in percentage; model 8). For brevity, we report only the coefficients of interest.

Table B.1 reports the estimates of the reduced-form eq. 5 using fixed effects. We look at the impact of board gender composition on the company-reported GPG to compare with estimates from table 4. Looking at the fullest specification (model 8 in table B.1), a higher female-director share is associated with a lower median gender pay gap that year. A 1 percentage-point increase in the female-director share is associated with a 0.03 percentage-point reduction in the gender pay gap. Replicating the same example that we discussed in section 4.1, this means that women go from 90.30p to 90.33p per £1 of men's earnings. This is an increase of 0.03 pence per £1 (a roughly 0.033% rise in female pay relative to male at that baseline).

2SLS vs. Fixed Effects In levels, the estimates using fixed effects are smaller. This means that for a woman earning £50,000 per annum, under the fixed effects, the increase is about £16.6

per year in relative terms when female directors increase by 1 p.p. Hence, the 2SLS estimate (specification 8, table 4) is about £7.2 higher - roughly 43% larger[22]- than the reduced-form FE estimate for the same 1 p.p. change in the share of female directors.

B.2 Regressions, by subsample

Table B.2 runs the fullest specification (specification 8 of tables 4 and B.1) in three subsamples. For the first two sub-samples, similar to Ahamed et al. (2019), employers with fewer (more) than 5,000 employees are identified as large (extra large) employers. For the third sub-sample, we restrict the analysis to the companies with insider shareholders.[23] Gupta and Sachdeva (2019) find that companies with inside investors perform better when using data from hedge funds on the financial performance of firms.

Table B.2: Effects of female directors on GPG; no outliers, by subsamples

	Dep. var.: Median difference of hourly pay between men and women/100							
	Full sample		Employers with less than 5,000 employees		Employers with more than 5,000 employees		Insider shareholders	
	IV (1)	FE (2)	IV (3)	FE (4)	IV (5)	FE (6)	IV (7)	FE (8)
Share of current female directors	-0.043^{***}	-0.030^{***}	-0.046^{***}	-0.033^{***}	0.062	0.069^{**}	-0.117^{***}	-0.076^{***}
	(0.012)	(0.009)	(0.013)	(0.009)	(0.050)	(0.031)	(0.030)	(0.020)
F-statistic for IV in first stage	26,699.2		25,622.4		741.5		3,110.5	
N	26,677	26,677	25,541	25,541	1,136	1,136	3,017	3,017
R2 adj.	0.318	0.318	0.314	0.314	0.434	0.434	0.236	0.237
FE: Firm	Yes	Yes	Yes	Yes	Yes	Yes	Yes	Yes
FE: LAD x year x 2-digit SIC	Yes	Yes	Yes	Yes	Yes	Yes	Yes	Yes

* $p < 0.1$, ** $p < 0.05$, *** $p < 0.01$

Note: Robust s.e. clustered at company level. Additional controls in all specifications: Logarithm of turnover per employee, Firm age, Employer size, Share of female employees, Profit per employee, Liquidity ratio, Logarithm of number of current directors (board size). For brevity, we report only the coefficients of interest. IV refers to our Bartik-type IV and reports estimates from the second-stage fitted values. FE refers to estimates from a fixed effects model (equation 5). Specification (1) replicates estimates from specification (8) of table 4. Specification (2) replicates estimates from specification (8) of table B.1.

Employer size When we restrict the sample to mid-sized and large firms (those with 250–5,000 employees), the coefficient of interest becomes more negative (around −0.05 in some specifications, vs. −0.04 for the full sample), indicating a larger impact of board diversity on the

[22] This is the 2SLS estimate relative to the fixed effects one, or

$$\frac{23.8 - 16.6}{16.6} = 0.434$$

[23] Insider shareholders are the directors (or senior officials) who are shareholders - usually they own more than 10% of the voting shares (Jensen and Meckling, 1976).

gap. In contrast, in an analysis focusing on the very largest companies (employing over 5,000), the effect is muted and not always statistically significant. This pattern suggests diminishing returns to board diversity in the absolute largest firms, possibly because these organizations are more complex or have formalized pay structures that leave less scope for board influence. It is worth noting here that the sample sizes are significantly different - only 5% of the sample includes extra-large employers.

Finally, a stronger effect for female directors exists in companies with insider shareholders regardless of the estimator used.

B.3 Bartik-IV approach: Exclude firm *i* and its corresponding sector

This section replicates the Bartik-IV approach. It differs from the main analysis, because when calculating the average share of current female directors we exclude both firm i and its corresponding 2-digit SIC sector $s(i)$. We then replicate regressions in table 4. To better illustrate the changes when excluding both the firm and its sector, we rewrite equation 6 as follows:

$$\overline{\text{share of female directors}_{-i,-s(i),t,r(i)}} \tag{B.1}$$

This changes equation 7 as follows:

$$g_{i,t,r(i)} = \frac{\overline{\text{share of female directors}_{-i,-s(i),t,r(i)}}}{\overline{\text{share of female directors}_{-i,-s(i),\text{base year},r(i)}}} \tag{B.2}$$

Table B.3 outlines the results of the second-stage when excluding outliers at 1% as in main analysis. We note that the significance and direction of results go towards the same direction. However, the impact of female directors on GPG is stronger in this - more conservative IV - approach.

Table B.3: Effects of female directors on GPG; 2SLS; second-stage estimates

	Dep. var.: Median difference of hourly pay between men and women							
	(1)	(2)	(3)	(4)	(5)	(6)	(7)	(8)
Share of current female directors (fitted)	-0.038**	-0.014	-0.008	-0.030**	-0.030**	-0.030**	-0.047***	-0.047***
	(0.015)	(0.015)	(0.014)	(0.015)	(0.015)	(0.015)	(0.015)	(0.015)
F-statistic for IV in first stage	12,302.8	11,925.1	6,674.5	6,281.3	6,278.5	6,277.8	6,159.9	6,158.5
N	26,677	26,677	26,677	26,677	26,677	26,677	26,677	26,677
R2 adj.	0.066	0.084	0.303	0.308	0.308	0.308	0.318	0.318
FE: Firm	No	No	Yes	Yes	Yes	Yes	Yes	Yes
FE: LAD x year x 2-digit SIC	No	No	Yes	Yes	Yes	Yes	Yes	Yes
FE: LAD	Yes	Yes	No	No	No	No	No	No
FE: Year	Yes	Yes	No	No	No	No	No	No

* $p < 0.1$, ** $p < 0.05$, *** $p < 0.01$

Note: Robust s.e. clustered at Local Authority level (models 1-2) and at company level (models 3-8). Additional controls by specification: Logarithm of turnover per employee (models 2-8), Firm age (models 3-8), Employer size (models 4-8), Share of female employees (models 4-8), Profit per employee (models 5-8), Liquidity ratio (model 6-8), Logarithm of number of current directors (board size; models 7-8), Return on total assets (in percentage; model 8). For brevity, we report only the coefficients of interest. First-stage estimates are available in table B.6.

B.4 Our Bartik-type instrument

We employ a Bartik (1991)-type instrument. We assume that changes in the trend of female directorships are exogenously given by the average fraction of female directors in an ITL1 region in a given year. The algorithm used to estimate and define the instrument is detailed in the main text (see equation 8 and its modification in Appendix B.3). Here, we present evidence for the relevance condition and exclusion restriction of the instrument. We report the first-stage estimates of the 2SLS models reported in the main text. Finally, we offer some methodological robustness checks: balance and pre-trend tests at the shift level, and placebo tests.

B.4.1 Relevance and Exclusion Restriction

The share of female directors aggregated at the ITL-1 region is expected to be correlated with the share of current female directors in each firm and year (*instrument is relevant*). Table B.4 shows the strong correlation between the instrument and the firm-level share of current female directors in 2019, 2020, and 2021.

Table B.4: Instrument and share of current female directors, OLS

	Share of current female directors in		
	2019	2020	2021
Instrument $\left(\widetilde{z_{i,t}}\right)$	0.834***	0.629***	0.439***
	(0.004)	(0.007)	(0.005)
N	8,643	4,320	6,360
R2 adj.	0.869	0.66	0.519

p < 0.1, ** p < 0.05, *** p < 0.01
Notes: Robust standard errors in parenthesis.

However, the share of female directors aggregated at the ITL-1 region is not correlated with any time-varying heterogeneity that could affect the wage determination and the board of directors at the firm level (*instrument is exogenous*). The exogeneity assumption is even stronger when we exclude not only firm i, but also its corresponding 2-digit SIC sector, $s(i)$, before aggregating the share of female directors in ITL-1 (see appendix B.3). Appendix B.3 takes into account both the cultural differences within regions and any sector-specific elements that affect female appointment on boards.

A reasonable comment would be to aggregate the "share" at the 2-digit SIC sector level instead of the ITL-1 region. We think this violates the exogeneity condition, as the sector may be correlated with the firm-level wage determination (Card et al., 2023).

B.4.2 First-stage estimates

Table B.5 presents the first-stage estimates of the Two-Stage Least Squares regression that employs our Bartik-type instrument as defined in equation 8.

Table B.5: Effects of female directors on GPG (instrument excludes company *i*); 2SLS, first-stage

	Dep. var.: Share of current female directors							
	(1)	(2)	(3)	(4)	(5)	(6)	(7)	(8)
$\tilde{z}$	0.615***	0.610***	0.558***	0.545***	0.545***	0.615***	0.540***	0.540***
	(0.007)	(0.007)	(0.006)	(0.006)	(0.006)	(0.007)	(0.006)	(0.006)
N	26677	26677	26677	26677	26677	26677	26677	26677
R2 adj.	0.695	0.698	0.657	0.664	0.664	0.695	0.668	0.668
F-statistic	50,359.1	48,803.4	28,723.8	27,173.9	27,161.8	27,161.2	26,701.4	26,699.2
Prob>F	0.000	0.000	0.000	0.000	0.000	0.000	0.000	0.000
FE: Firm	No	No	Yes	Yes	Yes	Yes	Yes	Yes
FE: LAD x year x 2-digit SIC	No	No	Yes	Yes	Yes	Yes	Yes	Yes
FE: LAD	Yes	Yes	No	No	No	No	No	No
FE: Year	Yes	Yes	No	No	No	No	No	No

* $p < 0.1$, ** $p < 0.05$, *** $p < 0.01$

Note: Robust s.e. clustered at Local Authority level (models 1-2) or company level (models 3-8). Additional controls by specification: Logarithm of turnover per employee (models 2-8), firm age (models 3-8), Employer size (models 3-7), Share of female employees (models 4-8), Profit per employee (models 5-8), Liquidity ratio (model 6-8), Logarithm of number of current directors (board size; models 7-8), Return on total assets (in percentage; model 8). Specifications 3-8 include interacted fixed effects.

Table B.6 presents the first-stage estimates of the Two-Stage Least Squares regression that employs the Bartik-type instrument for which we exclude both firm *i* and its corresponding 2-digit SIC sector (see appendix B.3).

B.4.3 Balance, pre-trend and placebo tests

We evaluate whether the identifying variation is plausibly orthogonal to pre-existing regional characteristics. Specifically, when identification relies on quasi-exogenous shocks regionally aggregated, it is essential to verify that the resulting regional treatment measure is not systematically correlated with any initial economic conditions. This is because it could confound the estimated effects. Hence, we show that regional trends are exogenous. Further, we show that our Bartik-type instrument does not predict any board-of-directors unrelated outcomes.

Table B.6: Effect of female directors on GPG (instrument excludes both company *i* and its 2-digit SIC sector); 2SLS; first-stage

	Dep. var.: Share of current female directors							
	(1)	(2)	(3)	(4)	(5)	(6)	(7)	(8)
$\tilde{z}$	0.304***	0.298***	0.221***	0.212***	0.212***	0.212***	0.208***	0.208***
	(0.061)	(0.060)	(0.027)	(0.027)	(0.026)	(0.026)	(0.026)	(0.026)
N	26,677	26,677	26,677	26,677	26,677	26,677	26,677	26,677
R2 adj.	0.398	0.409	0.427	0.448	0.448	0.448	0.457	0.457
F-statistic	12,302.8	11,925.1	6,674.5	6,281.3	6,278.5	6,277.8	6,159.9	6,158.5
Prob>F	0.000	0.000	0.000	0.000	0.000	0.000	0.000	0.000
FE: Firm	No	No	Yes	Yes	Yes	Yes	Yes	Yes
FE: LAD x year x 2-digit SIC	No	No	Yes	Yes	Yes	Yes	Yes	Yes
FE: LAD	Yes	Yes	No	No	No	No	No	No
FE: Year	Yes	Yes	No	No	No	No	No	No

* $p < 0.1$, ** $p < 0.05$, *** $p < 0.01$

Note: Robust s.e. clustered at Local Authority level (models 1-2) and at company level (models 3-8). Additional controls by specification: Logarithm of turnover per employee (models 2-8), Firm age (models 3-8), Employer size (models 4-8), Share of female employees (models 4-8), Profit per employee (models 5-8), Liquidity ratio (model 6-8), Logarithm of number of current directors (board size; models 7-8), Return on total assets (in percentage; model 8). For brevity, we report only the coefficients of interest.

Balance test We begin by testing for balance at the shift level by regressing the pre-period growth in the share of female directors on lagged regional economic indicators. These covariates —average weekly basic pay, total employment, gross value added, and the employment rate — capture a range of potential predictors of both wages and leadership representation.

Formally, we test:

$$g_{t,r} = \alpha + \beta X_{r,t-1} + \lambda_t + \mu_r + u_{r,t}$$

where $X_{r,t-1}$ denotes each lagged regional covariates in turn or in combination, λ_t are year fixed effects, and μ_r are regional fixed effects. If the instrument were systematically correlated with such predetermined factors, we would expect significant coefficients on β. Table B.7 reports the results.

Each lagged regional covariate is essentially zero and statistically insignificant when entered one-at-a-time or when used jointly. Therefore, there is no systematic correlation with pre-existing regional conditions. The balance test supports the as-good-as-random assumption for the regional component of our instrument.

Pre-trend test To assess whether regions differentially exposed to the instrument were already on diverging trajectories before the policy shocks, we conduct a shift-level pre-trend test. We regress pre-treatment growth in the share of female directors on lagged values of our key controls aggregated at the regional level: average turnover, employment, firm age, profit per

Table B.7: Balance test for our Batrik-type instrument; shift level

	Dep. var.: Regional growth of the share of female directors				
	(1)	(2)	(3)	(4)	(5)
Average weekly basic pay$_{t-1}$	0.012				0.012
	(0.008)				(0.007)
Average number of employees$_{t-1}$		-0.000			-0.000
		(0.000)			(0.000)
GVA$_{t-1}$			-0.000		0.000
			(0.000)		(0.000)
Employment rate$_{t-1}$				0.041	0.046
				(0.055)	(0.055)
N	36	36	36	36	36
R2 adj.	0.895	0.898	0.881	0.877	0.911
FE: Year	Yes	Yes	Yes	Yes	Yes
FE: Region	Yes	Yes	Yes	Yes	Yes

* $p < 0.1$, ** $p < 0.05$, *** $p < 0.01$

Notes: Robust standard errors clustered at the regional level. Regional data for the average weekly basic pay, number of employees, gross value added, and employment rate come from ONS Nomis (accessed on 5 May 2025).

employee, liquidity ratio, and within-firm average wages.

Table B.8 reports the results of the pre-trend test. Results are not significant for most of the variables, confirming no pre-trend issues at the shift level.

Placebo tests Here, we implement three placebo tests of the Bartik-type instrument, using firm-level outcomes that are unrelated to board gender composition given FAME data availability. First, we use *net interest* (thousand GBP), which is largely determined by existing borrowing terms. Second, we proxy the *effective cash tax rate* as cash taxes paid (thousand GBP) divided by profit (or loss) before tax (thousand GBP). This should be driven by statute and short-run profitability rather than female directorships. Third, using firm filing dates, we construct an indicator equal to one if the firm submits its accounts on a different month than in the previous period and zero otherwise. This administrative housekeeping should not move with board gender composition.

All regressions include the full set of controls from the main specification. Table B.9 reports only the coefficient on the variable of interest. Because these outcomes are sparsely populated in FAME, the sample size falls substantially relative to the main regressions. Across all spec-

Table B.8: Pre-trend test for our Bartik-type instrument; shift-level

	Dep. var.: Regional growth of the share of female directors
Turnover_{t-1}	0.379
	(0.766)
$\text{Number of employees}_{t-1}$	0.000
	(0.000)
Firm age_{t-1}	0.049
	(0.040)
$\text{Profit per employee}_{t-1}$	0.000
	(0.000)
$\text{Liquidity ratio}_{t-1}$	0.804*
	(0.440)
$\text{Wages and salaries}_{t-1}$	0.000
	(0.000)
N	36
R2 adj.	0.119

* $p < 0.1$, ** $p < 0.05$, *** $p < 0.01$

Notes: Robust standard errors clustered at the regional level. All independent variables are the average at the ITL-1 regional level 1-time lag variable.

ifications, the second-stage coefficient on the share of female directors is not statistically significant. Therefore, there is no evidence that our instrument shifts other firm outcomes. This suggests our exclusion restriction is credible.

Table B.9: Effects of female directors on firm-level unrelated to BoD outcomes; 2SLS; second-stage estimates

	Dependent variables:		
	Net interest (1)	Effective cash tax rate (2)	Change of month accounts date (3)
Share of current female directors	−10,241.439	−1.677	0.023
	(22,330.794)	(1.749)	(0.025)
F-statistic for IV in first stage	12,065.2	12,065.2	12,065.2
N	10,910	10,910	10,910
R2 adj.	-0.041	-0.142	-0.165
FE: Firm	Yes	Yes	Yes
FE: LAD x year x 2-digit SIC	Yes	Yes	Yes

* $p < 0.1$, ** $p < 0.05$, *** $p < 0.01$

Note: Robust s.e. clustered at the company level. Outcomes derived from FAME and are described in the appendix text. Additional controls: logarithm of turnover per employee, firm age, Employer size, Share of female employees, Profit per employee, Liquidity ratio, Logarithm of number of current directors (board size), Return on total assets (in percentage). For brevity, we report only the coefficients of interest. All models have the same first-stage equation.

C Comparing our wage distribution to ASHE wage distribution

C.1 ASHE data and sample description

In the subsequent sections, we compare our wage distributions to the wage distribution derived from administrative firm-level survey data. We provide supplementary information about the datasets employed and our methodology for constructing subsamples for firms with at least 250 employees. The UK Data Service offers publicly accessible documentation and variable descriptions associated with the Annual Survey of Hours and Earnings (ASHE; Office for National Statistics (2023)). The ONS releases address the data quality and consistency of the ASHE.

C.2 Why do we compare to ASHE?

In the main body of the paper, we outline our approach to estimate the natural logarithm of the average wage by worker gender within each firm (section 2.2.4). Our estimation assumes that (i) the wage distributions for both men and women follow a log-normal distribution. (ii) Further, the ranking of women and men in the overall pay distribution within a firm is determined based on the known proportion of women and men in each pay quartile. Finally, we use the annual average remuneration per employee within each firm as available in FAME. In what follows, we call the distribution derived in the main paper GEO/FAME. To facilitate comparability, we aim to plot this estimated wage distribution alongside the wage distribution derived from the Annual Survey of Hours and Earnings (ASHE). For consistency, we will restrict our ASHE analysis to companies with a minimum of 250 employees. Since ASHE surveys a random sample of all PAYE employees (further description follows), workers in companies present in GEO/FAME are very likely to be questioned.

C.3 ASHE: data description

We focus on the period from 2017 to 2021. During this timeframe, the ASHE aims to represent a random sample of all employees, regardless of occupation or employer size. Due to the legal obligation of employers to report payroll information, the ASHE exhibits a high response rate and is considered to be accurate. There is no cumulative attrition from the panel, as individuals who are not included in the ASHE in a particular year, for any reason, remain part of the sampling frame for the subsequent year. Assuming a 100% response rate, the ASHE constitutes a true one percent random sample of employees based on their National Insurance Number, specifically those with a numerical part ending in 14. However, there are two main sources

of under-sampling, both due to lack of current tax records. This may happen if (a) individuals recently changed jobs or (b) earn very little (primarily part-time workers) and are exempt from paying income tax or National Insurance during the period when their employers respond to the questionnaire. Starting from 2004, the ASHE aimed to address the under-sampling issue by including supplementary responses for individuals without a PAYE reference and making efforts to capture employees whose jobs changed between the sampling frame determination in January and the reference period in April. ONS has stated that the biases stemming from these amendments were small, hence their impact would not significantly affect our results. The ASHE also introduced imputations by using similar matched observations from other respondents as ”donors” to fill in missing data, such as basic hours of work. One can use these imputations for weighting purposes, but in this section, we present solely non-weighted results.[24]

C.4 Employee-Employer matched data

We utilise the ASHE annual cross-sections for the years 2017 to 2021 to construct a panel dataset. Our approach involves establishing connections between employees across successive years based on their unique identifiers. By employing this method, we are able to retroactively assign enterprise reference numbers (entrefs) for missing data points, starting from 2021. This is feasible because the ASHE dataset includes a variable indicating whether an employee has remained in the same job as in the previous reference period. It is important to note, however, that relying solely on this “same job” variable does not allow us to differentiate between job changes within firms and job changes across firms. Once we have linked two consecutive years using this methodology, we employ local unit identifiers to impute missing entrefs for individuals within the same year. The Office for National Statistics (ONS) clarifies that local unit identifiers are not consistent across years but are designed to identify establishments within a given year. We extend this procedure to update missing entrefs for all relevant years, up to and including 2017.

[24]At this stage, we opt to disregard any weighting considerations since they are primarily intended to ensure that aggregate results are representative of the population in terms of worker characteristics (i.e., age, gender, occupation and region of work) to match the Labour Force Survey and are not specific to the firm level analysis we are currently focusing on.

C.5 Sample construction

To ensure the reliability and consistency of our analysis, we apply several filters to the dataset, which are in line with the methodology used in the ONS's annual publication on ”Patterns of Pay” using the ASHE. The filters we employ are as follows:

1. retain only observations only for individuals aged 16 to 64.

2. exclude individuals who have been flagged as having experienced a loss of pay in the reference period due to absence, employment starting in the period, or short-time working.

3. exclude trainees and apprenticeships by dropping observations that are not marked as being on an adult rate of pay.

4. remove observations with missing values for basic hours, gross weekly earnings, or hourly wage rates. Basic hours represent the ordinary working hours for an employee in a standard week, excluding overtime and meal breaks. Gross weekly pay is the primary recorded value in the survey, with overtime pay deducted from it. Hourly rates are then derived by dividing gross weekly earnings by basic hours worked.

5. eliminate observations where the number of basic hours worked is over 100 or less than 1. These extreme values could be indicative of measurement errors or the inclusion of overtime, which we aim to avoid.

6. exclude the top and bottom 1% of the pay distributions to mitigate the influence of outliers on our analysis.

7. Define full-time employment as working over thirty basic hours in a week. However, we acknowledge that there may be a few discrepancies in some years, particularly related to teaching contracts, where the ONS applies a different definition. We address these discrepancies by recoding all observations to apply the thirty-hour threshold consistently.

In order to construct the *sample of large firms*, i.e., those with at least 250 employees, we utilize the information available in the ASHE dataset, specifically the number of employees listed for each enterprise in the Inter-Departmental Business Register (IDBR). The following steps are taken to create the large firms sample:

1. We exclude employers whose exact enterprise reference number of employees, as recorded in the IDBR, is less than 250. This ensures that we focus on enterprises with the similar size as in GEO/FAME data.

2. We remove observations where the IDBR status, number of employees, or industry classification is missing. This ensures that we have complete and reliable information for the selected firms.

For the annual pay analysis, we apply similar filtering criteria for constructing the sample of large firms, with some additional considerations:

1. In addition to the aforementioned filters, we exclude observations where the employee is reported to not have been in the same job for 12 months.

2. We drop observations with zero or missing values for annual gross pay.

When working with the ASHE annual cross-section datasets, we apply the same approach as described above. However, in this case, we can utilise a unique enterprise-level identifier available in the dataset to identify firms within each year.

C.6 Wage distributions

Below, we plot the wage distributions for ASHE and GEO/FAME by gender. For ASHE, I use the sample of large employers (with at least 250 employees). Figure C.1 below plots the distribution of natural log of annual wage excluding the top and bottom 1% of the distribution. As far as GEO/FAME is concerned, we plot the distribution of average wages for men and women that we estimated in the main part of the paper based on the annual average wages and salaries of employees. Solid lines replicate figure 2 of the main paper.

Figure C.1a plots ASHE with no weights, while figure C.1b plots ASHE with simple sample weights. For each year, the weight is the outcome of the number of observations for a given company over the total number of observations (ONS requires us to drop companies with less than 10 observations in the sample). In expectation, the mean value for male distributions and female distributions between GEO/FAME and ASHE should be the same. We find the same average value for women, but not for men. Our method under-predicts the GEO/FAME mean for male wages, or, GEO/FAME data understate male earnings.

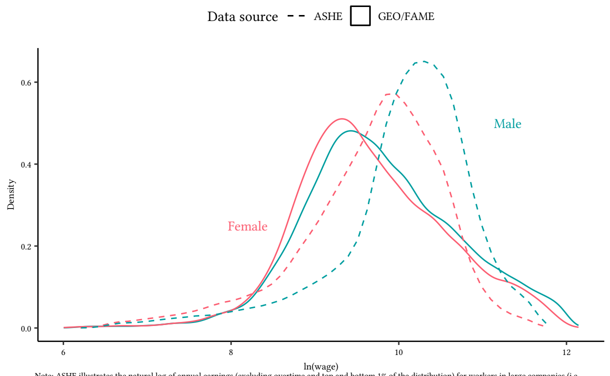

Note: ASHE illustrates the natural log of annual earnings (excluding overtime and top and bottom 1% of the distribution) for workers in large companies (i.e. employers with at least 250 employees) between 2017-2021. GEO/FAME distributions plot the natural log of average wages as derived and used in the paper based on the average worker wages and salaries between 2017-2021. Distributions do not use any weight. All distributions use epanechnikov kernel and 0.8 bandwidth for comparability purposes.
Source: Authors' elaboration based on gender-pay-gap-service.gov.uk, FAME and ONS ASHE.

(a) ASHE (with no weights)

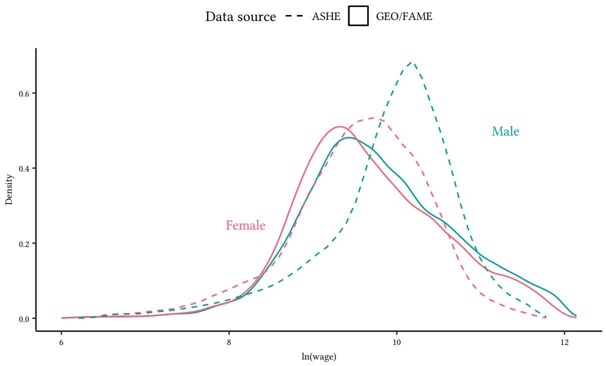

Note: ASHE illustrates the natural log of annual earnings (excluding overtime and top and bottom 1% of the distribution) for workers in large companies (i.e. employers with at least 250 employees) between 2017-2021. GEO/FAME distributions plot the natural log of average wages as derived and used in the paper based on the average worker wages and salaries between 2017-2021. ASHE distribution uses simple sample weights, i.e. the number of observations for each company over the total number of observations within a year. All distributions use epanechnikov kernel and 0.8 bandwidth for comparability purposes.
Source: Authors' elaboration based on gender-pay-gap-service.gov.uk, FAME and ONS ASHE.

(b) ASHE (simple sample weights)

Figure C.1: Wage distributions, by gender and data source

C.6.1 Why is there an inconsistency?

Our analysis is correct if and only if (a) wages are similarly defined between GEO/FAME and ASHE and (b) company-reported GPG is accurate.

Regarding point (a), there may be a difference in the definition of wages between FAME and ASHE. We are not certain what elements of wages and salaries are included in FAME. Our ASHE estimates include basic pay excluding overtime.

Regarding point (b), Bailey et al. (2022) find that around 5% of employers provide gender statistics that are mathematically implausible, indicating a prevalent occurrence of misreporting, whether intentional or unintentional. This may significantly drive the observed differences in the distribution of male wages.

Finally, our observed distributional differences may be associated with the ASHE data composition. Forth et al. (2022) find that there is systematic attrition in ASHE which may introduce bias when using the data. They find that male employees, younger employees, and those with low tenure appear to have higher than expected rates of attrition in ASHE. Comparing ASHE to the Annual Population Survey (APS), they find that 25% of employees drop out in a given year from ASHE, but only 8% from the APS. The response rate of ASHE may further affect these differences. Forth et al. (2022) find that ASHE responses correspond to 60-66% of ONS estimated jobs at the end of each financial year.

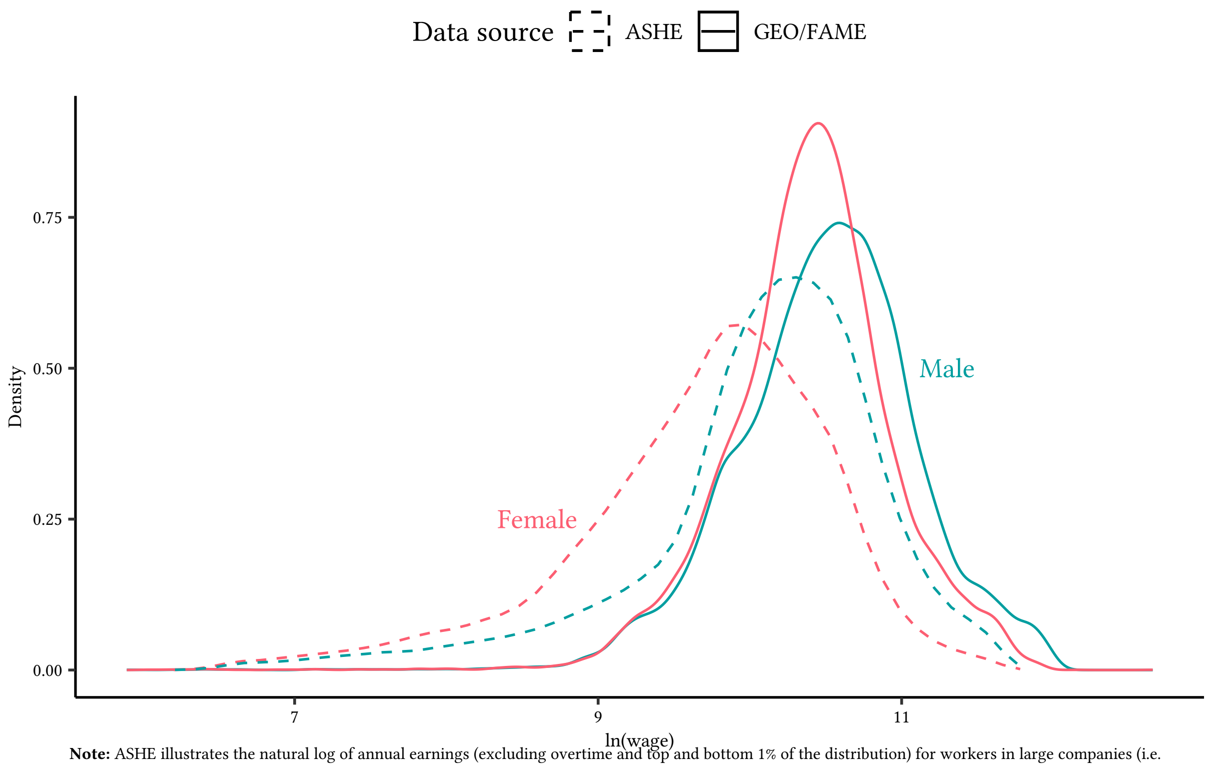

Note: ASHE illustrates the natural log of annual earnings (excluding overtime and top and bottom 1% of the distribution) for workers in large companies (i.e. employers with at least 250 employees) between 2017-2021. GEO/FAME distributions plot the natural log of average wages as derived and used in the paper based on the average worker remuneration between 2017-2021. None distribution uses any weight. All distributions use epanechnikov kernel and 0.8 bandwidth for comparability purposes.
Source: Own elaboration based on ASHE, FAME and gender-pay-gap.service.gov.uk

Figure C.2: Wage distributions, by gender and data source; alternative definition of wages for GEO/FAME

C.6.2 Alternative wage distribution for GEO/FAME

Figure C.2 plots the distribution of the log of annual wage excluding the top and bottom 1% of the distribution for ASHE without any weight. As far as GEO/FAME is concerned, we plot the distribution of average wages for men and women that we estimated in the main part of the paper based on the annual average worker remuneration.
